# Factors Affecting the Outcome of Scleral Buckling Surgery for Primary Rhegmatogenous Retinal Detachment

**DOI:** 10.1155/2018/9016302

**Published:** 2018-11-13

**Authors:** Ritesh Shah, Raghunandan Byanju, Sangita Pradhan, Sudha Ranabhat

**Affiliations:** ^1^MD Ophthalmology, Bharatpur Eye Hospital, Bharatpur-10, 44207 Chitwan, Nepal; ^2^Chief Medical Director and Head of Department, Department of Retina, Bharatpur Eye Hospital, Bharatpur-10, Chitwan, Nepal; ^3^MD Ophthalmology Resident, Bharatpur Eye Hospital, Bharatpur-10, 44207 Chitwan, Nepal

## Abstract

**Introduction:**

Scleral buckle surgery retains a special place in treatment of retinal detachment despite development of new and advanced vitreoretinal surgical techniques. The outcome of any retinal detachment surgery depends on correct selection of patient, type and nature of detachment, and the expertise. This study aims to evaluate various other parameters that determine the outcome of scleral buckle surgery.

**Method:**

Records of 55 patients with primary rhegmatogenous retinal detachment treated with scleral buckling over a duration of 18 months that had a minimum of 3-month follow-up were retrospectively reviewed. Preoperative and postoperative characteristics were recorded. Parameters that were evaluated to determine the outcome were best-corrected visual acuity (BCVA), anatomical success, and complications.

**Results:**

A total of 51 eyes of 50 patients that met the inclusion criteria were included. Mean age was 41 ± 19.9 years (range: 9 to 83). Primary anatomical success was achieved in 80.4%. Parameters significantly associated with the anatomical outcome of surgery were status of lens, preoperative visual acuity, and extent of retinal detachment. There was a significant improvement of visual acuity postoperatively.

**Conclusion:**

Scleral buckle surgery is a highly effective surgery in uncomplicated retinal detachment cases, and single surgery success rates are better in cases with good preoperative visual acuity, partial detachment, and clear crystalline lens.

## 1. Introduction

The first segmental scleral sponge exoplant surgery was performed by Ernst Custodis over 6 decades ago [1]. The modern scleral buckling technique came into practice after Charles Schepens [2] discovered binocular indirect ophthalmoscope with a scleral depressor, and Harvey Lincoff [3] started the use of cryotherapy.

Scleral buckling may be technically challenging and require prolonged surgical time but is usually associated with low risk of ocular complications, less expense, and has similar outcome when compared with pars plana vitrectomy [4, 5].

Excellent surgical technique, correct selection of patient, and nature of retinal detachment are equally essential for good surgical outcome. However, in scleral buckling surgery, there are numerous other factors that determine the outcome.

The aim of this study is to evaluate the result of scleral buckle surgery with special emphasis on the influence of different parameters on postoperative anatomical and visual outcome.

## 2. Materials and Methods

A retrospective review of 55 consecutive medical files of all the patients who presented with primary rhegmatogenous retinal detachment and treated with scleral buckling between July 2016 and December 2017 was conducted. Patients with a minimum 3-month follow-up were included in the study. Exclusion criteria included tractional or exudative RD, advanced proliferative vitreoretinal changes (grade C and D) where vitrectomy was required, diabetic retinopathy, significant cataract obscuring view of fundus details, choroidal detachment, and those with history of globe laceration repair. Fifty-one eyes of 50 patients fulfilling the inclusion and exclusion criteria were included in the study. The study was approved by the Institutional Review Board.

Medical records were reviewed to obtain data on age, sex, duration of symptoms, lens status, location and quadrants of retinal detachment, number of retinal breaks, status of macula (on or off), type of scleral buckle used (circumferential or segmental), intraoperative injection of temporary vitreous adjunct (gas/air), anatomic outcome of surgery, need for additional surgery, pre- and postoperative best-corrected visual acuity (at day one, 2 weeks, 6 weeks, and 3 months), pre- and postoperative IOP, and postoperative refractive error.

### 2.1. Surgical Technique

All the surgeries were performed under peribulbar block or general anesthesia (in children and uncooperative patients). At the beginning of surgery, the ocular surface was washed with 5% povidone iodine. A 360° conjunctival peritomy with dissection of tenon capsule was done. Traction sutures were placed beneath the insertions of the exposed rectus muscle with 4/0 silk. Transscleral cryotherapy was used to treat the breaks. A silicone band (silicone band 240) and sleeve were used for circumferential buckling by passing it beneath the 4 rectus muscles and scleral tunnels. The buckling materials either segmental or circumferential (silicone tire; 276/279) were sutured to the sclera with 5/0 polyester sutures. A scleral incision was made from the side of the horizontal rectus muscle in accordance with the location of the detachment and subretinal fluid drainage was performed. Air or 0.3 cc of pure perfluoropropane gas was used in eyes requiring internal tamponade. The conjunctiva was closed with 8/0 Vicryl sutures.

### 2.2. Statistical Analysis

For statistical purpose, best-corrected visual acuity was measured using the Snellen chart, and the measurements were converted to logMAR. A modified scale was used to assign nonnumerical visual acuities to logMAR scores of 1.7, 2.0, 2.3, and 3.0 for “counting fingers”, “hand movements”, “perception of light”, and “no perception of light,” respectively [6].

Data analysis was performed using SPSS software (version 20, SPSS Inc., Chicago, IL, USA). Frequencies and percentages were computed to describe categorical data. Chi-square test and independent *t* test was used to make comparisons, as appropriate. Paired *t* test was used to compare mean preoperative and postoperative visual acuity. *p* value <0.05 was considered significant.

## 3. Results

Fifty-one eyes of 50 patients including 68.6% male and 31.4% female who fulfilled the inclusion criteria were assessed at Bharatpur eye hospital, Chitwan, Nepal. Demographic details are presented in Table 1.

### 3.1. Anatomical Outcome

Primary anatomical success with single surgery was achieved in 80.4% patients. Significantly better anatomical outcome was observed in phakic patients (*p*=0.03) and patients with better preoperative visual acuity (*p*=0.006). Presence of total retinal detachment was associated with poor anatomical outcome (*p*=0.04). On the contrary, gender, duration of symptom, positive history of trauma, number of breaks, intraoperative use of intravitreal C3F8 or air, and status of macula were not significantly associated with the final anatomical outcome (Table 2).

### 3.2. Visual Outcome

Regarding functional outcome, there was a significant improvement of visual acuity after scleral buckle surgery including cases with residual detachment. (*p* < 0.001). Postoperative visual outcome according to duration of symptom was not statistically significant. (Table 3). Although the status of macula had no significant effect on anatomical outcome, postoperative visual acuity was remarkably better in macula on retinal detachment cases (*p*=0.003). Mean BCVA in the macula on and off cases was 0.41 logMAR and 1.1 logMAR, respectively.

### 3.3. Complications

Postoperatively, temporary rise in IOP above 21 mm of Hg was seen in 17 eyes (33.3%) and all were adequately controlled by topical antiglaucoma medication. The mean age of patients with postoperative raised IOP was less (33.9 years) than those with normal IOP (44.5 years) but was not statistically significant (*p*=0.075). Similarly the use of intravitreal gas or air was not significantly associated with raised IOP (*p*=0.27). Other complications included vitreous hemorrhage (1.96%) and buckle infection (1.96%).

## 4. Discussion

The rate of anatomical success in the present series was 80.4%. This was consistent with the MUSTARD study, one of the largest studies done on scleral buckling surgery in Europe [7]. However, other studies have reported a success rate of over 90% [8, 9].

Factors that influenced the anatomical outcome in this study were lens status, preoperative visual acuity, and extent of retinal detachment. Pseudophakic retinal detachment was significantly associated with poor anatomical outcome in this series. This was similar to many other studies [10, 11]. On the contrary, Custodis [1] and Scharwtz [12] have reported no difference in anatomical outcome owing to lens status. Vukosavljević et al [13] explained that the poor outcome in pseudophakic retinal detachment was because of poor visualisation of retina and localisation of retinal break due to capsular opacities.

Retinal detachment with single retinal break is associated with better anatomical outcome [14–16]. However, in this series, the number of breaks was not associated with the difference in anatomical outcome. Seven of 38 eyes (18.4%) with single retinal break had persistent detachment as compared with 3 of 10 eyes (23%) with more than one break.

Results from the MUSTARD study have confirmed favorable outcome in the younger age group (21–30 years) and poor in the extreme age group (<10 and >70 years) In this series, no significant difference was observed in the rate of retinal detachment in different age groups, which was similar to a longitudinal study done by Hassan et al. [17] A significant improvement in BCVA after scleral buckling surgery was observed in this study. The macula on retinal detachment cases had better visual outcome. Although it is an established fact that visual outcome mainly depends on the duration of macular detachment, our study could not determine the significance of the duration of symptoms in predicting postoperative visual outcome. This could be due to late presentation (mean duration of symptoms at presentation-16.6 weeks), imprecise reporting of the duration of symptoms in the pediatric age group and small sample size. Other studies have shown that functional recovery was best when surgery was performed within 1 week of onset of symptoms [9, 14].

Elevated IOP after scleral buckling surgery has been reported ranging from 3.3% to 16% [18–20]. Postoperative rise in IOP in this series was remarkably high (33.3%) compared with previous studies. However, all the cases were adequately controlled by topical antiglaucoma medication. Almost half of the cases of postoperative rise in IOP was seen in the younger age group (<30 years). The mean age of patients with raised IOP was lesser than those with normal IOP, however, not statistically significant (*p*=0.07).

## 5. Conclusion

Scleral buckle is a safe and effective surgery for uncomplicated primary rhegmatogenous retinal detachment. Postoperative anatomical and visual outcomes are best achieved in cases with clear crystalline lens, good preoperative visual acuity, and partial retinal detachment.

## Figures and Tables

**Table 1:** Demographics of 51 patients who underwent scleral buckle surgery for retinal detachment.

Variables	Values
Gender *n* (%)	
** **Male	34 (68.6%)
** **Female	16 (31.4%)
Mean age in years, x ± sd (range)	41 ± 19.9 (range: 9 to 83)
Age in years, *n*(%)	
** **<30	17 (34%)
** **30–50	8 (16%)
** **>50–65	17 (34%)
** **>65	8 (16%)
Affected eye, *n* (%)	
** **Right	30 (59%)
** **Left	21 (41%)
Lens status	
** **Phakic	35 (68.6%)
** **Pseudophakic	16 (31.4%)
History of ocular trauma, *n*(%)	
** **Yes	11 (21.6%)
** **No	
Duration of symptom in weeks, x ± sd (range)	16.6 ± 44.7 (range: 0.3–240)
Status of macula	
** **On	8 (15.7%)
** **Off	43 (84.3%)
Total retinal detachment, *n* (%)	12 (23.5%)
Number of breaks	
** **1	38 (74.5%)
** **2	12(23.5%)
** **3	1(1.9%)
Intravitreal C3F8/air use	
** **Yes	23 (45.1%)
** **No	28(54.9%)
Anatomical outcome	
** **Attached	41 (80.4%)
** **Residual RD	10 (19.6%)
BCVA of the affected eye, x ± sd	
** **At presentation	1.45 ± 0.71 logMAR (range: 0–2.3 logMAR)
** **Postoperative 3 months	0.98 ± 0.6 logMAR(range: 0.17–2.3 logMAR)

**Table 2:** Anatomical outcome of sclera buckling surgery according to preoperative clinical characteristics.

	Total	Retina attached	Persistent detachment	*p* value
Number of eyes	51	41	10	
Age (x ± sd, years)	41 ± 19.9	40.15 ± 19.9	44.4 ± 20.4	0.54^a^
** **<30 years	17	15	2	
** **30–50 years	16	13	3	0.69^b^
** **51–65 years	14	10	4	
** **>65 years	4	3	1	
Sex (male/female)	34/16	28/13	7/3	0.619^b^
Duration of symptom (x ± sd, weeks)	16.6 ± 44.7	15.15 ± 46.6	22.71 ± 37.2	0.63^a^
Preoperative BCVA (x ± sd)	1.45 ± 0.71 logMAR	1.32 ± 0.73	2 ± 0.28	0.006^a^
Total retinal detachment (*n*)	12	7	5	0.04^c^
Positive history of trauma(*n*)	11	10	1	0.42^c^
Number of break (1/≥2) (*n*)	38/13	31/10	7/3	0.7^c^
Macula status (on/off) (*n*)	43	8/33	0/10	0.32^c^
Intravitreal C3F8/air use	23	20	3	0.43^b^
Lens status (phakic/pseudophakic) (*n*)	35/16	31/10	4/6	0.03^b^

^a^ *t* test. ^b^Chi-square test. ^c^Fisher's exact test.

**Table 3:** Final anatomical outcome 3 months after scleral buckling in patients with anatomical success according to duration of symptom.

Duration of symptom (days)	Patients (*n*, %) BCVA <0.5	BCVA ≥0.5	*p* value
1–7	5	6	0.18
8–30	5	10	
>30	2	13	
Total	12	29	

## Data Availability

The datasets generated during and/or analysed during the current study are available from the corresponding author on reasonable request.
